# Implementation and Evaluation of a Breast Cancer Disease Model Using Real-World Claims Data in Germany from 2010 to 2020

**DOI:** 10.3390/cancers16081490

**Published:** 2024-04-13

**Authors:** Dominik Dannehl, Alexandra von Au, Tobias Engler, Léa Louise Volmer, Raphael Gutsfeld, Johannes Felix Englisch, Markus Hahn, Sabine Hawighorst-Knapstein, Ariane Chaudhuri, Armin Bauer, Markus Wallwiener, Florin-Andrei Taran, Diethelm Wallwiener, Sara Yvonne Brucker, Stephanie Wallwiener, Andreas Daniel Hartkopf, Tjeerd Maarten Hein Dijkstra

**Affiliations:** 1 Department of Women’s Health, Tübingen University, 72076 Tübingen, Germany; tobias.engler@med.uni-tuebingen.de (T.E.); gutsfeldr@gmail.com (R.G.); johannes.englisch@med.uni-tuebingen.de (J.F.E.); markus.hahn@med.uni-tuebingen.de (M.H.); armin.bauer@med.uni-tuebingen.de (A.B.); diethelm.wallwiener@med.uni-tuebingen.de (D.W.); sara.brucker@med.uni-tuebingen.de (S.Y.B.); andreas.hartkopf@med.uni-tuebingen.de (A.D.H.); tjeerd.dijkstra@med.uni-tuebingen.de (T.M.H.D.); 2 Department of Gynecology and Obstetrics, Heidelberg University, 69120 Heidelberg, Germany; alexandra.vonau@med.uni-heidelberg.de; 3 AOK Baden-Wuerttemberg, 70188 Stuttgart, Germany; sabine.knapstein@bw.aok.de (S.H.-K.); dr.med.ariane.chaudhuri@bw.aok.de (A.C.); 4 Department of Gynecology, Halle University, 06120 Halle, Germany; markus.wallwiener@uk-halle.de; 5 Department of Gynecology and Obstetrics, Freiburg University, 79106 Freiburg im Breisgau, Germany; florin-andrei.taran@uniklinik-freiburg.de; 6 Department of Obstetrics and Perinatal Medicine, Halle University, 06120 Halle, Germany; stephanie.wallwiener@uk-halle.de; 7 Institute for Translational Bioinformatics, University Hospital Tübingen, 72076 Tübingen, Germany

**Keywords:** breast cancer, real-world evidence, claims data, hormone receptor, HER2, overall survival, distant recurrence-free survival

## Abstract

**Simple Summary:**

This research analyzed health data from 27,869 female breast cancer patients and 55,738 controls in Germany to develop a breast cancer disease model, focusing on stages and tumor subtypes from 2010 to 2020. It found that the majority of patients had HR+ tumors, with HR+/HER2− being the most common subtype. The study revealed significant survival differences across stages and subtypes, with stages B and C showing much lower survival rates than early-stage or control groups. It also noted worse outcomes for the HR−/HER2− subtype. This is the first study of its kind to utilize German claims data to model breast cancer, offering crucial insights into the disease’s real-world epidemiology and treatment outcomes.

**Abstract:**

Breast cancer is the leading cause of cancer-related mortality among women in Germany and worldwide. This retrospective claims data analysis utilizing data from AOK Baden-Wuerttemberg, a major statutory German health insurance provider, aimed to construct and assess a real-world data breast cancer disease model. The study included 27,869 female breast cancer patients and 55,738 age-matched controls, analyzing data from 2010 to 2020. Three distinct breast cancer stages were analyzed: Stage A (early breast cancer without lymph node involvement), Stage B (early breast cancer with lymph node involvement), and Stage C (primary distant metastatic breast cancer). Tumor subtypes were estimated based on the prescription of antihormonal or HER2-targeted therapy. The study established that 77.9% of patients had HR+ breast cancer and 9.8% HER2+; HR+/HER2− was the most common subtype (70.9%). Overall survival (OS) analysis demonstrated significantly lower survival rates for stages B and C than for controls, with 5-year OS rates ranging from 79.3% for stage B to 35.4% for stage C. OS rates were further stratified by tumor subtype and stage, revealing varying prognoses. Distant recurrence-free survival (DRFS) analysis showed higher recurrence rates in stage B than in stage A, with HR−/HER2− displaying the worst DRFS. This study, the first to model breast cancer subtypes, stages, and outcomes using German claims data, provides valuable insights into real-world breast cancer epidemiology and demonstrates that this breast cancer disease model has the potential to be representative of treatment outcomes.

## 1. Introduction

In women, breast cancer is the most common cancer type and the leading cause of cancer-related deaths in Germany and worldwide [1,2]. Breast cancer is divided into different subtypes according to hormone receptor (HR) and HER2 receptor expression. Currently, breast cancer treatment is based on breast surgery, radiotherapy, and adjuvant endocrine therapy in hormone receptor-positive (HR+) patients and, where appropriate, (neo)adjuvant chemotherapy and HER2-targeted antibody treatment [3,4].

To characterize the clinical and epidemiological components of breast cancer in a real-world setting, different data sources can be utilized: retrospective reviews of clinical cases, retrospective state-wide, national, or international cancer registries, prospective clinical trials, and prospective clinical registries [5,6,7,8,9,10]. However, retrospective claims data analysis has emerged as an important tool to further elucidate patient characteristics, comorbidities, treatment algorithms, and clinical outcomes [11,12,13]. Furthermore, claims data analysis represents an important tool for estimating the economic burden of specific diseases and assessing the cost-effectiveness of certain treatments [14,15]. Claims data aggregate socioeconomic, epidemiological, and clinical data from different healthcare providers in the inpatient and outpatient setting, allowing a holistic evaluation of treatment courses.

In Germany, every citizen is legally required to be covered by a statutory or private health insurance plan. To date, 96 statutory health insurances and 52 private health insurances operate in this country [16,17]. One of the largest statutory health insurances is the AOK Baden-Wuerttemberg, which insures 4.6 million policyholders [18].

Since claims data do not cover information about breast cancer tumor biology or tumor stage, they have not been used as a tool to analyze outcome parameters in Germany. Thus, the aim of this work was to implement and evaluate a breast cancer disease model using claims data from AOK Baden-Wuerttemberg.

## 2. Materials and Methods

This study is a retrospective claims data analysis from a large statutory German health insurance provider (AOK Baden-Wuerttemberg). The study was conducted according to the guidelines of the Declaration of Helsinki and was approved by the Ethics Committee of Tuebingen University (380/2020BO).

AOK Baden-Wuerttemberg provided an anonymized dataset consisting of 97,121 patients—95,499 women and 1622 men—who received a breast cancer diagnosis (ICD10 code C50) and 94,849 age-matched control patients—93,253 women and 1596 men—without a breast cancer diagnosis between 2010 and 2020 (inclusive) [19]. The dataset consisted of 15 tables (see Supplementary Table S1 for details). Patient deaths were reported until 31 May 2022.

All patients included in the analysis received inpatient treatment for invasive breast cancer (C50) between 1 July 2010 and 31 December 2019 (analysis period) or were control group subjects. Due to unreliable encoding of C50 (breast cancer) in the outpatient setting, patients were excluded if the dataset did not contain concomitant C50 diagnoses in the inpatient setting. Patients were also excluded if the first encoding for invasive breast cancer ranged between 1 January 2010 and 30 June 2010 since the onset of invasive breast cancer could not be clearly identified. Due to a short or missing follow-up period, patients with C50 diagnosis were excluded if the first encoding for invasive breast cancer ranged between 1 January 2020 and 31 December 2020.

Moreover, patients were excluded from the analysis if the overall insurance duration was less than 40% of the observation period. The observation period ranged from 1 January 2010 until 31 December 2020 if the patient did not die. The observation period ranged from 1 January 2010 until the time of death for those patients who died, except for those patients who died after 31 December 2020, for whom we took 31 December 2020 as the end of the observation period. For 90% of patients, this observation period consisted of the 11 years between 1 January 2010 and 31 December 2020 (alive patients) or 1 January 2010 until death (dead patients). Further exclusion criteria were the encoding of secondary neoplasia before or after the first diagnosis of invasive breast cancer (except non-melanoma skin cancer, ICD code C44), the onset of distant metastatic disease at least 6 months prior to the first encoding of C50, and male sex (Table 1). Patients were excluded from the control group if no data for the insurance period were available, if the insurance period comprised less than 40% of the observation period, if patients developed neoplastic disease (except non-melanoma skin cancer, ICD code C44), or developed distant metastatic disease within the observation period, and male sex (Table 1).

Clinical and pathological breast cancer stages after the TNM and UICC classification of malignant tumors are not encoded in German claims data [20]. We identified three distinct breast cancer stages grossly resembling the established UICC stages: (i) Stage A: early breast cancer without pathological axillary lymph node involvement (encoding of C50 without encoding of C77.3); (ii) Stage B: early breast cancer with pathological axillary lymph node involvement (encoding of C77.3 within 6 months of breast cancer diagnosis date); and (iii) Stage C: primary distant metastatic breast cancer (appearance of distant metastatic disease within 6 months of breast cancer diagnosis date). Distant metastatic disease could be encoded during an inpatient treatment or in more than two consecutive calendar quarters in the outpatient setting. Distant metastatic disease was defined as the encoding of C77–C79, except C77.3 (axillary lymph node involvement) and C77.9 (lymph node involvement, not otherwise specified). Since the onset of distant metastatic disease could be missed due to inadequate encoding, the first diagnosis of distant metastatic disease could also be defined by: (i) administration of chemotherapy for at least five calendar quarters (Supplementary Table S2), the start of metastatic disease being defined as the middle of the fifth quarter that chemotherapy was administered; (ii) HER2-targeted antibody therapy for at least seven calendar quarters (Supplementary Table S2), the start of metastatic disease being defined as the middle of the seventh quarter that antibody therapy was administered; (iii) medication intake that defines distant metastatic disease (Supplementary Table S2) for at least two quarters, the start of metastatic disease being defined as the middle of the first quarter that therapy was administered; and (iv) histological examination of putative malignant lesions (e.g., biopsies, paracentesis) (Supplementary Table S2) [21], the start of metastatic disease being defined as the date of histological examination.

Since German claims data do not encode breast cancer biology, the histologic subtype was reconstructed from the systemic therapy the patient received. This medication was identified by Anatomic Therapeutic Chemical (ATC) codes [22]. ATC codes represent filled prescriptions and, thus, the medication a patient receives at the pharmacy and not necessarily the prescribed medication. Patients were defined as either HR- or HER2-positive if they received the corresponding medication at least once in the observation period after the first diagnosis of C50 (Supplementary Table S2). Since GnRH analogs in premenopausal patients can be used to protect ovarian function during chemotherapy, GnRH analogs themselves were not sufficient for defining HR+.

Distant recurrence-free survival (DRFS) was defined as the time between the first encoding of C50 in an inpatient setting and the first diagnosis of distant metastatic disease. Overall survival (OS) was determined as the time between the first encoding of C50 in an inpatient setting and death. Breast cancer surgery was assessed using operational and procedure key numbers (OPS; see Supplementary Table S2) [21].

Matching was performed to pair each patient with breast cancer with two unique patients in the control group (1:2 ratio). Since age at first diagnosis is an important predictor for OS, year of birth was selected as the main criterion. As the second matching criterion, a “no exclusion before diagnosis” constraint was applied. This consistency constraint excludes control patients from a match when (a) they died or (b) they canceled insurance before the C50 diagnosis of the breast cancer patients. In detail, the matched controls should be insured and live longer than the breast cancer diagnosis date of their match since the breast cancer patients were insured and survived at least until the date of their breast cancer diagnosis.

Matching was implemented using the R package optmatch. This package uses network flow algorithms [22] to find the matches that minimize the age difference between breast cancer patients and controls. The “no exclusion before diagnosis” constraint was implemented as a caliper distance matrix with value infinity when a control patient’s (a) date of death or (b) cancellation of insurance was reported before the matched breast cancer diagnosis and otherwise with a value 0 [23]. This “no exclusion before diagnosis” caliper distance matrix was added to the birth year difference matrix and given to the optmatch function pairmatch() for optimal matching. Due to technical limitations in processing the large data set on a computer with 32 GB RAM, the problem was split into four equal parts as follows: included patients were numbered consecutively (ID 1 to 27,869 for breast cancer patients and ID 27,870 to 104,965 for control patients). These IDs were assigned a “match cohort” based on the remainder of integer division by 4 of their ID number. Optimal matching was then run for each match cohort independently, and resulting matches were combined. The results of optimal matching are shown in Supplementary Table S3. There are two matches with a 7-year birth year difference. This is due to a breast cancer patient who was born in 1912 and still alive as of May 2022, for which there were no closely matching controls (within two years of birth year difference). In both these cases the matching algorithm could not find two matching controls within two years of birth year difference.

Data processing and statistical analysis were performed using R (version 4.3.0, R Core Team (2023)) and RStudio (Version 2023.06.1+524, Posit PBC, Boston, MA, USA). We used the packages optmatch [24], ggsurvfit 0.3.0, and survival 3.3.5. Furthermore, we used patchwork 1.1.3, gt 0.9.0, janitor 2.2.0, lubridate 1.9.2, forcats 1.0.0, stringr 1.5.0, dplyr 1.1.2, purr 1.0.1, readr 2.1.4, tidyr 1.3.0, tibble 3.2.1, ggplot2 3.4.2, and tidyverse 2.0.0. Kaplan-Meier methodology was utilized to estimate OS and DRFS together with the standard deviation (SD). We used the log-rank test function survdiff() from package survival with the default values to compare survival curves.

## 3. Results

Of 97,121 patients with breast cancer in the dataset, 69,252 (71.3%) were excluded (Table 1). In all, 52% (50,098/97,121) of patients were excluded due to C50 diagnosis only in the outpatient setting, which we deemed unreliable as no inpatient treatment, such as concomitant breast surgery or systemic therapy, was performed in the observational period. Furthermore, 9.9% (9640/97,121) of all patients were excluded because they developed secondary neoplasia before or after the first diagnosis of breast cancer. When the first diagnosis of breast cancer was not made within the time window (06/2010 to 01/2020), 7.3% (7069/97,121) of all patients were excluded. The remainder of patients were excluded due to the onset of metastatic disease before breast cancer was diagnosed (1487/97,121; 1.5%), an insurance period below 40% of the observation period (652/97,121; 0.7%), death before the first encoding of C50 (91/97,121; 0.1%), or male sex (215/97,121; 0.2%). In total, 27,869 female patients (28.7%) with breast cancer could be included and were subjected to further analysis. In the control group, 10,964/94,809 (11.6%) patients were excluded because they developed a secondary neoplasia. Moreover, 2594/94,809 (2.7%) were excluded as their insurance period was below 40% of the observation period. Another 1138/94,809 (1.2%) of all patients were excluded due to male sex. As we chose to match one patient with breast cancer to two control patients, 55,738/94,809 (58.8%) of patients in the control group were included and hence subject to further analysis (Table 1).

Table 2 highlights the age distribution and place of residence. Due to age matching, both the breast cancer and the control cohort show the same age pattern. Of the patients, 16.0% (4467/27,869 breast cancer, 8934/55,738 controls) were below 50 years of age at breast cancer diagnosis, whereas 21.4% (5975/27,869 breast cancer, 11,950/55,738 controls) were diagnosed in their fifties, 22.7% (6331/27,869 breast cancer, 12,662/55,738 controls) in their sixties and seventies (6321/27,869 breast cancer, 12,642/55,738 controls), respectively, and 17.1% (4775/27,869 breast cancer, 9550/55,738 controls) of patients were at least 80 years of age. In all, 25.6% (7133/27,869) of patients from the breast cancer cohort lived in rural areas, 32.0% (8928/27,869) in suburban areas, and 42.3% (11,792/27,869) in urban areas. A balanced distribution pattern can be observed in the control group, where 25.4% (14,139/55,738) lived in rural areas, 31.6% (17,609/55,738) in suburban areas, and 42.8% (23,831/55,738) in urban areas.

Table 3 displays the estimated baseline patient characteristics that were reconstructed using claims data as described in the Materials and Methods section. Here, 77.9% (21,697/27,869) of patients showed HR+ breast cancer and 9.9% (2747/27,869) HER2+ breast cancer. The most common tumor subtype was HR+/HER2− (19,767/27,869; 70.9%), followed by HR−/HER2− (5355/27,869; 19.2%), HR+/HER2+ (1930/27,869; 6.9%), and HR−/HER2+ (817/27,869; 2.9%). Most patients (18,892/27,869; 67.8%) were assigned to stage A (early breast cancer without pathologic axillary lymph node involvement). In all, 4732/27,869 patients (17.0%) displayed early breast cancer with pathologic axillary lymph node involvement, and 4245/27,869 patients (15.2%) showed primary metastatic breast cancer. Of all patients, 80.1% (22,337/27,869) received breast surgery, 58.9% (16,425/27,869) radiation therapy, and approximately one-third systemic therapy (9182/27,869; 32.9%).

**Figure 1:**
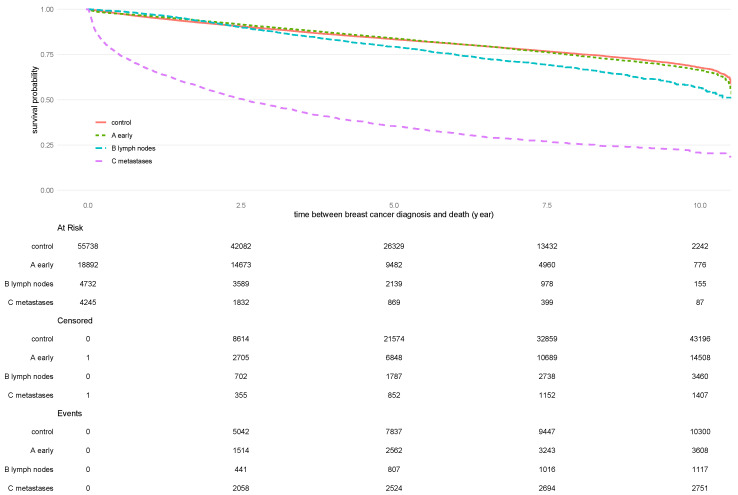
Overall survival stratified according to tumor stage.

OS for patients assigned to stage A was not significantly different from the control group. However, OS was significantly lower in stages B and C than in the control group (*p* < 0.001 for each) (Figure 1). Mean 5-year OS for patients assigned to the control group was 83.4% ± 0.2%, 84.0% ± 0.3% for stage A, 79.3% ± 0.7% for stage B, and 35.4% ± 0.8% for stage C. Mean 10-year OS was 67.7% ± 0.4% for the control group, 66.3% ± 0.7% for stage A, 56.6% ± 1.5% for stage B, and 20.9 ± 1.0% for stage C (for details, see Supplementary Table S4).

Kaplan–Meier analysis of patients with stage A breast cancer (green dotted line), stage B breast cancer (blue dotted line), and stage C breast cancer (violet dotted line). Patients from the control group are depicted in red. The shadowed area in each color highlights the 95% confidence interval.

**Figure 2:**
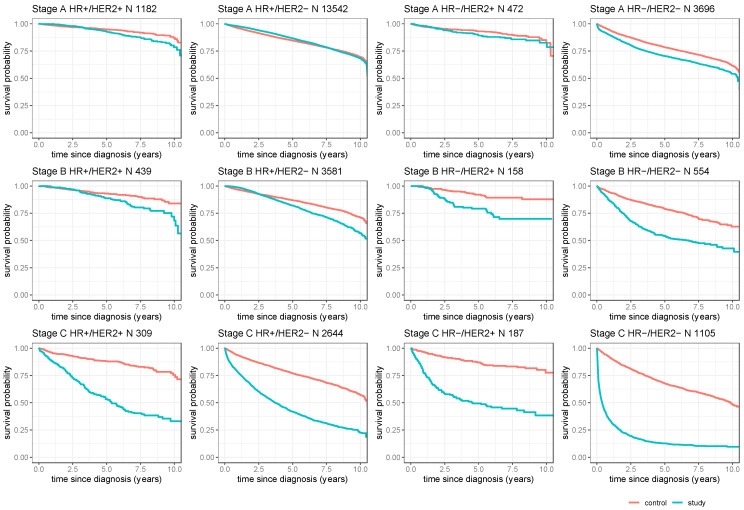
Overall survival stratified according to tumor stage and estimated tumor subtype.

Figure 2 displays OS rates stratified by estimated tumor subtype and disease stage. The OS rate for breast cancer patients with HR−/HER2+ breast cancer in stage A was not significantly different from the respective subtype-specific control group (see Supplementary Table S5). The mean 5-year OS was 89.6% ± 1.6% for the subtype-specific control group. Breast cancer patients with HR+/HER2− breast cancer in stage A showed a significantly better OS rate than did its subtype-specific control group (*p* < 0.001, Supplementary Table S5). The mean 5-year OS was 86.7% ± 0.3% for HR+/HER2− breast cancer in stage A and 84.7% ± 0.2% for the subtype-specific control group. However, in patients with HR−/HER2− and HR+/HER2+ breast cancer, OS was significantly worse than in the subtype-specific control group in stage A (*p* < 0.001, Supplementary Table S5). The mean 5-year OS was 70.5% ± 0.8% for HR−/HER2− breast cancer in stage A and 78.5% ± 0.5% for the subtype-specific control group. For HR+/HER2+ breast cancer patients in stage A, the mean 5-year OS was 93.0% ± 0.9% and 94.8% ± 0.5% for the subtype-specific control group.

In stage B, all breast cancer subtypes showed significant differences in OS rate compared to its subtype-specific control group (*p* < 0.001, Supplementary Table S5). The Mean 5-year OS was 88.8% ± 1.7% for HR+/HER2+ breast cancer in stage B and 93.1% ± 0.9% for the subtype-specific control group; for HR+/HER2− breast cancer patients in stage B, the mean 5-year OS was 82.1% ± 0.7% and 87.0% ± 0.4% for the subtype-specific control group; for HR−/HER2+ breast cancer patients in stage B, the mean 5-year OS was 79.2% ± 3.5% and 92.1% ± 1.7% for the subtype-specific control group; and for HR−/HER2− breast cancer patients in stage B, the mean 5-year OS was 54.1% ± 2.3% and 79.0% ± 1.4% for the subtype-specific control group.

In patients with primary metastatic breast cancer (stage C), regardless of the estimated tumor subtype displayed, the OS rate was significantly worse than for their respective subtype-specific control group (*p* < 0.001, Supplementary Table S5). The mean 5-year OS rate for every subgroup was at least 35% lower than its subtype-specific control group. Patients with HR+/HER2+ breast cancer in stage C displayed a mean 5-year OS of 53.2% ± 3.2% compared to 88.1% ± 1.4% for the subtype-specific control group. In the HR+/HER2− cohort in stage C, the mean 5-year OS was 41.8% ± 1.1% and 77.0% ± 0.6% for the subtype-specific control group. In patients with HR−/HER2+ breast cancer in stage C, the mean 5-year OS was 49.4% ± 3.9% and 86.9 ± 1.9% for the subtype-specific control group. Patients with HR−/HER2− breast cancer in stage C show the worst OS: the mean 5-year OS was 12.7% ± 1.1% and 67.8% ± 1.1% for the subtype-specific control group.

Kaplan-Meier analysis on overall survival of patients with different estimated tumor subtypes and stages. Patients in the breast cancer group are depicted in green; the subtype-specific age-matched control group is depicted in red. The shadowed area in each color highlights the 95% confidence interval. The three panels on the left side show HR+/HER2+ breast cancer patients, the three panels in the middle left show HR+/HER2− breast cancer patients, the three panels in the middle right display HR−/HER2+ breast cancer patients, and the three panels on the right side show HR−/HER2− breast cancer patients in three different stages. Across all estimated tumor subtypes, the patients show the best overall survival in stage A, followed by stage B, and the worst overall survival in stage C. Patients with HR−/HER2− breast cancer show the worst overall survival rates in all stages.

**Figure 3:**
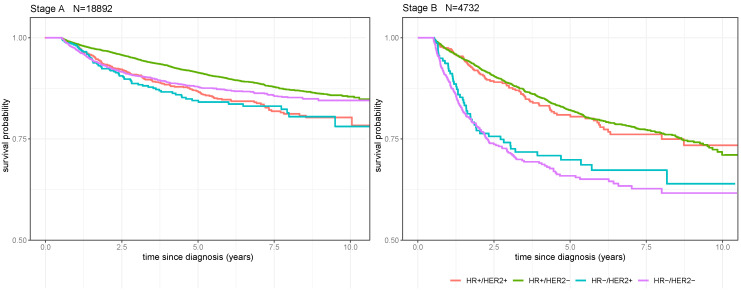
Distant recurrence-free survival stratified according to tumor stage and estimated tumor subtype.

DRFS was defined as the time between breast cancer diagnosis and the first diagnosis of distant metastatic disease. The comparison to a control group was not applicable in this analysis. Thus, HR+/HER2+ breast cancer, the estimated tumor subtype with the best prognosis, was set as a reference for statistical comparison. In stage A, the DRFS rate was not significantly different for HR−/HER2+ and HR−/HER2− breast cancer compared to HR+/HER2+ breast cancer (Figure 3, Supplementary Table S6). However, HR+/HER2− breast cancer showed a significantly better DRFS rate than did HR+/HER2+ breast cancer in stage A. Mean 5-year DRFS in stage A was 86.7% ± 1.1% for HR+/HER2+ breast cancer, 91.4% ± 0.3% for HR+/HER2− breast cancer, 84.5% ± 1.9% for HR−/HER2+ breast cancer, and 87.9% ± 0.6% for HR−/HER2− breast cancer.

In patients with pathologically involved axillary lymph nodes (stage B), the probability of disease recurrence was higher. Only patients with HR+/HER2− breast cancer had a comparable risk of disease recurrence. The other estimated subtypes had a significantly worse DRFS compared to HR+/HER2+ breast cancer in stage B (HR−/HER2+ *p* < 0.01; HR−/HER2− *p* < 0.001, Supplementary Table S6). The mean 5-year DRFS was 80.9% ± 2.1% for HR+/HER2+ breast cancer, 82.1% ± 0.7% for HR+/HER2− breast cancer, 69.9% ± 3.9% for HR−/HER2+, and 65.9% ± 2.3% for HR−/HER2− breast cancer in stage B.

Kaplan-Meier analysis on distant recurrence-free survival of patients with different estimated tumor subtypes and stages. For the sake of clarity, the *y*-axis was capped at 0.5. The left panel shows patients with stage A breast cancer; the right panel shows patients with stage B breast cancer. HR+/HER2− breast cancer patients are depicted in red, HR+/HER2+ breast cancer patients in green, HR−/HER2+ breast cancer patients in blue, and HR−/HER2− breast cancer patients in violet. The shadowed area in each color highlights the standard deviation. In stage A, patients with HR−/HER2− breast cancer show the worst 5-year distant recurrence-free survival rates. The 5-year distant recurrence-free survival rates are comparable for the other tumor subtypes. In stage B, HR+/HER2+ breast cancer shows the best 5-year distant recurrence-free survival rate, followed by HR+/HER2− breast cancer, HR−/HER2+ breast cancer, and HR−/HER2− breast cancer.

## 4. Discussion

This retrospective analysis demonstrates the feasibility of constructing an accurate breast cancer disease model utilizing real-world claims data. Comparable DRFS and OS data for specific breast cancer subtypes were observed when compared to the current literature [25,26,27,28,29,30,31,32,33]. However, compared to international claims data sets, claims data in Germany lack critical information regarding tumor histology, biology, grading, precise TNM stage, treatment-related adverse effects, and patient-reported outcome measures [34,35]. In our analysis, approximately 70% of all breast cancer cases were excluded from further analysis. Most of these were cases for which the ICD10 diagnosis C50 was coded only in an outpatient setting during the observational period: Neither did these patients receive breast cancer-specific therapy (surgery, endocrine therapy, chemotherapy, or radiation therapy), nor were they referred to a hospital for inpatient treatment. Hence, we concluded that a breast cancer diagnosis was presumed in these patients, which could not be confirmed. In Germany, patients primarily visit a general practitioner or general gynecologist in private practice who refers them to breast cancer specialists for further diagnosis and treatment. If one of these doctors in private practice detects any unusual palpation or imaging findings, they document a suspected diagnosis of breast cancer (C50). However, this diagnosis is frequently not confirmed after the patient is referred to a specialist. Another possible explanation for this observation is that the treatment for breast cancer has been conducted before the beginning of the observational period, and the patients visit the general gynecologist for follow-up care. Thus, ICD10 C50 diagnoses that were only recorded in the outpatient setting were deemed unreliable.

To exclusively evaluate the impact of breast cancer diagnosis on OS rates, we excluded patients in whom a secondary neoplasm was recorded before or after their breast cancer diagnosis during an inpatient setting or in two different quarters in the outpatient setting from the analysis. Additionally, allowing for secondary neoplasms after the initial breast cancer diagnosis would have made it impossible to evaluate the impact of breast cancer on DRFS, as the neoplasm that led to distant metastatic disease could not be determined. The code C44 represents the only exception as it encompasses “non-melanoma skin cancer”, which comprises basal cell carcinoma (75%), squamous cell carcinoma (25%), and rare neuroendocrine Merkel cell carcinoma of the skin (<1%) [1]. In 2018, roughly 95,000 new cases of non-melanoma skin cancer were diagnosed in American women [1]. The diagnosis of non-melanoma skin cancer is typically associated with older age, as the median age at diagnosis is 74 years. Additionally, this illness has a negligible effect on relative survival rates and rarely triggers distant metastatic disease [1,36]. If C44 had been an exclusion criterion, another 7.6% of the 27,869 patients in the experimental group and another 6.3% of the 55,738 control patients included would have been excluded.

Tumor subtypes were estimated using the type of the prescribed medication. Overall, the estimated tumor subtype in this analysis closely matched those reported in the current literature. However, in our analysis, the proportion of triple-negative breast cancer was higher, while the proportion of HR+ and HER2+ breast cancer was lower when compared to real-world data [6,7,10]. This observation can be explained by primary nonadherence to endocrine therapy and HER2-targeted therapy since patients might be refraining from filling the prescription for endocrine therapy at the pharmacy or have refused to undergo systemic therapy from the beginning [37]. Overestimation of the fraction of HR−/HER2− patients by patients who did not receive systemic therapy is consistent with the observation that this group was older and received less chemotherapy than would be expected from real-world datasets [6]. Regrettably, we were unable to develop a method to further classify these patient groups. While the subtype-specific DRFS rates of the estimated tumor subtypes were comparable to the existing literature, the subtype-specific OS rates were worse [25,27,30,38,39]. To address this issue, we used age as the matching factor, with each patient from the breast cancer group matched to two patients from the control group using the R package optmatch [24]. As a result, we were able to calculate breast cancer-specific mortality without the confounding effect of patient age. Thus, our model was able to accurately delineate breast cancer-specific mortality as a delta between the survival rates of the breast cancer cohort and the control group compared to the existing literature [25,27,30,38,39]. In primary metastatic breast cancer (stage C), the mean 5-year OS rate for HER2+ breast cancer in this analysis ranged from 49.0% to 53.0%, which is comparable to the median OS rate for HER2+ breast cancer patients in the literature [29]. As the majority of patients analyzed during the observation period between 2010 and 2020 did not receive a CDK 4/6 inhibitor, the mean 5-year OS rate of 41.8% among primary metastatic HR+/HER2− breast cancer patients is consistent with data published in the era before CDK 4/6 inhibitors were introduced [28,32,40,41]. However, our analysis shows that the mean 5-year OS rate for primary metastatic breast cancer in HR−/HER2− patients is only 13%. This is lower than the range of mean 5-year OS between 17.4 and 23.9 months that has been reported [32,33]. As outlined before, the analyzed cohort of HR−/HER2− patients included both true HR−/HER2− tumor biology and a significant number of patients with putative HR+ and/or HER2+ tumor biology who did not receive adequate breast cancer treatment. Consequently, DRFS and OS rates from this cohort cannot be compared directly to the published literature. Especially in stage C HR−/HER2− breast cancer (primary metastatic), OS declines rapidly within the first few months after breast cancer diagnosis. It is possible that a significant proportion of these patients are in worse general condition and are either unable or unwilling to receive adequate breast cancer therapy.

In our model, the tumor stage was determined from data concerning the involvement of pathological lymph nodes and the presence of distant metastatic disease. Although pathological lymph node involvement has a significant impact on recurrence and survival rates, the tumor stages reconstructed in our analysis cannot be precisely compared to established TNM and UICC stages, which also consider tumor size and the number of involved lymph nodes [20]. Especially the number of pathologically involved lymph nodes negatively correlates to distant recurrence-free survival and breast cancer-specific mortality [42,43,44,45]. However, recent research could demonstrate that the omission of axillary lymph node dissection after detection of 1–2 pathologically involved lymph nodes using sentinel lymph node biopsy does not impair the oncologic outcome [46,47,48]. The onset of metastatic disease was reliably captured in the dataset. However, further examination of individual cases revealed that the onset of metastatic disease using codes C77, except C77.3 and C77.9 or C78–79, was often later than the coding of medications or procedures that define a metastatic situation. Thus, the onset of distant metastatic disease could be accurately modeled by incorporating further criteria in the analysis. We defined the onset of primary or secondary distant metastatic disease as discovering at least one of the following criteria: (i) undergoing chemotherapy for a minimum of five calendar quarters, (ii) undergoing HER2-targeted antibody therapy for a minimum of seven calendar quarters, (iii) taking medications that define distant metastatic disease for a minimum of two quarters, or (iv) histologic examination of putative malignant lesions (e.g., biopsies, paracentesis).

These tumor-stage models approximately resemble the survival outcomes of breast cancer patients that are described in the literature [30]. However, 5-year OS rates for non-metastatic breast cancer were unfavorable compared to the literature [25,27,30,31,39]. As previously stated, the age at first diagnosis of breast cancer was identified as a confounding variable in the present analysis. Using age-matched control groups specific to breast cancer subtypes, our study revealed that patients with HR+/HER2− and HR−/HER2− breast cancer are significantly older at the time of their first breast cancer diagnosis than patients with HER2+ breast cancer and patients that were included in recent clinical studies. Interestingly, patients with HR+/HER2− early breast cancer in stage A show a significantly better mean 5-year OS than subtype-specific, age-matched control patients. The mean 5-year OS was 86.7% ± 0.3% for HR+/HER2− breast cancer and 84.4% ± 0.2% for its age-matched control group. This phenomenon might be attributed to survivorship bias [49]. As patients attend regular physician appointments, attention can be directed to other potential medical concerns, which can then be treated effectively.

Analyzing medical claims data is an effective means to study healthcare utilization and associated costs in a real-world setting. This method can elucidate how healthcare services are delivered, reveal disease prevalence patterns, and show the efficacy of treatments in routine clinical practice. Moreover, claims data offer a large sample size, making it a robust dataset for analysis. Claims data cover lengthy observation periods, facilitating longitudinal analysis of disease progression, treatment patterns, and long-term outcomes. Additionally, examining medical claims data can be a cost-effective alternative to conducting prospective clinical trials in specific circumstances. Additional data collection is often unnecessary and can save time and resources. Nonetheless, analyzing health insurance data has limitations, as claims data mainly concentrate on billing and administrative information, lacking detailed clinical data. Thus, indirect modeling of disease stages and tumor subtypes utilizing coding and prescription data can result in imprecise analyses. Although the inclusion and exclusion criteria were established prior to analyzing the dataset, these criteria had to be adjusted to accurately characterize the breast cancer and control cohorts [50,51].

Our claims data set included 4.6 million people with statutory health insurance from one of Germany’s largest health insurers [18]. Nevertheless, individuals with high annual incomes can opt for private health insurance and might thus be underrepresented in this dataset. Additional biases exist within the claims data as they are restricted to a specific region and lack information on external factors such as patient behavior, preferences, and social determinants of health [52]. Moreover, regulatory and incentive-driven influences on financial reimbursement can introduce a reporting bias, which was not assessed in this analysis [53]. Furthermore, claims data analyses do not reflect recent developments or changes in healthcare practices. It is, therefore, crucial to recognize that claims data must be analyzed critically and that the results cannot simply be generalized [54]. To enhance the consistency of the applied criteria, it is necessary to conduct random selection and manual analysis of individual cases.

## 5. Conclusions

This retrospective analysis of claims data from a major German health insurance provider represents the first attempt to introduce and assess a breast cancer disease model in Germany. By carefully selecting cases and matching patient age to a large control group, we showed that this breast cancer disease model is representative of treatment outcomes when compared to current clinical trials and real-world data analyses, especially when compared to HR-positive or HER2-positive patient populations [25,26,27,28,29,30,31,32,33]. Further analyses will focus on rare subgroups such as male breast cancer patients, comorbidities, adherence to treatment, and long-term effects of breast cancer treatment such as secondary diseases, fertility rates, and mental health.

## Figures and Tables

**Table 1:** Inclusions and exclusions.

	Breast Cancer		Control	
	N	%	N	%
Total	97,121	100	94,809	100
Excluded unreliable C50	50,098	51.6	0	0
Excluded diagnoses outside the time window	7069	7.3	0	0
Excluded secondary neoplasia	9640	9.9	10,964	11.6
Excluded metastases	1487	1.5	3017	3.2
Excluded insured too short	652	0.7	2594	2.7
Excluded death before diagnosis	91	0.1	0	0
Excluded male gender	215	0.2	1138	1.2
Excluded due to matching	0	0	21,358	22.5
Included	27,869	28.7	55,738	58.8

**Table 2:** Age and place of residence.

	Breast Cancer		Control	
	N	%	N	%
age at breast cancer diagnosis (years)				
<50	4467	16.0	8934	16.0
50ies	5975	21.4	11,950	21.4
60ies	6331	22.7	12,662	22.7
70ies	6321	22.7	12,642	22.7
>80	4775	17.1	9550	17.1
urban density level				
rural	7133	25.6	14,139	25.4
suburban	8928	32.0	17,609	31.6
urban	11,792	42.3	23,831	42.8
missing	16	0.1	159	0.3

**Table 3:** Estimated baseline patient characteristics.

	N	%
Estimated receptor expression		
HR+	21,697	77.9
HR−	6172	22.1
HER2+	2747	9.9
HER2−	25,122	90.1
Estimated biologic subtype		
HR+/HER2+	1930	6.9
HR+/HER2−	19,767	70.9
HR−/HER2+	817	2.9
HR−/HER2−	5355	19.2
Stage		
A	18,892	67.8
B	4732	17.0
C	4245	15.2
Breast surgery		
yes	22,337	80.1
no	5532	19.9
Radiation therapy		
yes	16,425	58.9
no	11,444	41.1
Systemic therapy		
yes	9182	32.9
no	18,687	67.1

## Data Availability

Data were provided by AOK Baden-Wuerttemberg. Due to privacy reasons and data security regulations, data are only available with the consent of AOK Baden-Wuerttemberg.

## Supplementary Materials

The following supporting information can be downloaded at https://www.mdpi.com/article/10.3390/cancers16081490/s1. Table S1: Characterization of the provided data set; Table S2: List of relevant ICD codes, ATC codes, and OPS codes; Table S3: Match quality for females; Table S4: 5- and 10-year overall survival rates stratified after disease stage; Table S5: 5-year overall survival rates stratified after disease stage and estimated tumor subtype; Table S6: 5-year distant recurrence-free survival rates stratified after disease stage and estimated tumor subtype.
