# Supplementary material for: Implementation and Evaluation of a Breast Cancer Disease Model Using Real-World Claims Data in Germany from 2010 to 2020
Source: Cancers (Basel). 2024 Apr 13;16(8):1490. doi: 10.3390/cancers16081490 (PMC11049278; doi:10.3390/cancers16081490)
Supplement: Supplementary file 1 [file cancers-16-01490-s001.zip › cancers-2939180-supplementary.pdf]

Supplementary Table S1: Characterization of the provided data set

	Description	NumPat	NumRec	Variables
1	Baseline	97121	97121	PatientID, BirthYear, Gender, InsuranceStatus, Schooling, Education, ZipCode, UrbanDensity, DeathYearMonth, StartDiagnosisC50YearMonth
2	Insurance period	97121	899324	PatientID, InsuranceYear, InsuranceDaysPerYear, CancellationReason
3	Insurance program	51187	82647	PatientID, Program, StartDate, EndDate, CancellationReason
4	Disease management program	31752	46613	PatientID, DMP_NR, Program, StartDate, EndDate
5	Hospital main ICD codes	86421	462302	PatientID, CaseID, YearQuarter, StartDate, EndDate, ICD1, ICD1txt, DRG1
6	Hospital secondary ICD codes	83151	2593705	PatientID, CaseID, YearQuarter, ICD2, ICD2txt
7	Hospital procedure OPS codes	83644	1451201	PatientID, CaseID, YearQuarter, OPS, OPStxt
8	Outpatient specialty (AGS) codes	97088	13843826	PatientID, CaseID, YearQuarter, AGS, AGStxt, Origin
9	Outpatient ICD codes	97082	58952841	PatientID, CaseID, YearQuarter, ICD, ICDtxt, Origin
10	Outpatient EBM codes	97088	90218288	PatientID, CaseID, YearQuarter, EBM, EBMtxt, Origin
11	Pregnancies ICD and EBM codes	2101	3012	PatientID, YearQuarter, BirthDate, ICD, EBM, Origin
12	Inability to work ICD codes	33225	386892	PatientID, CaseID, YearQuarter, StartDate, EndDate, AGS, ICD
13	Drug specialty (AGS) codes	96809	12154581	PatientID, YearQuarter, ReceiptID, AGS, AGStxt, ReceiptDate
14	Drug doses (DDD) and names (ATC)	96809	18130721	PatientID, YearQuarter, ReceiptID, PZN, PZNtxt, DDD, ATC, ATCtxt
15	Drug doses (DDD) and names (ATC)	23251	1663345	PatientID, YearQuarter, ReceiptID, PZN, PZNtxt, DDD, ATC, ATCtxt

NumPat = number of patients, NumRec = number of records, DMP = disease management program, AGS = key defining the physician group, DRG = disease-related groups, DDD = defined daily dose, PZN = pharmaceutical central number

Supplementary Table S2: List of relevant ICD codes, ATC codes, and OPS codes

**ICD**	
C50%	Breast Cancer
C77%	Lymph node metastasis
C77.3	Axillary lymph node metastasis
C77.9	Lymph node metastasis, not otherwise specified
C78%	Secondary malignant neoplasm of respiratory and digestive organs
C79%	Secondary malignant neoplasm of other and unspecified sites
**ATC chemotherapy**	
L01DB%	Anthracyclines
L01AA01	Cyclophosphamide
L01BC%	Antimetabolites
L01CD%	Taxanes
L01XA%	Platinum derivatives
**ATC palliative chemotherapy and targeted therapy**	
L01EF01<br>L01EF02<br>L01EF03	Palbociclib<br>Ribociclib<br>Abemaciclib
L01XK01<br>L01XK02<br>L01XK03<br>L01XK04<br>L01XK05	Olaparib<br>Niraparib<br>Rucaparib<br>Talazoparib<br>Veliparib
L01EM03	Alpelisib
L01EG02	Everolimus
L01XC07	Bevacizumab
L01CA%	Vinca alkaloids
L01XX41	Eribulin
**ATC endocrine therapy**	
L02BA%	SERM/SERD (Fulvestrant, Tamoxifen)
L02BG%	Aromatase inhibitors
L02AE%	GnRH analogues
**ATC HER2-targeted therapy**	
L01XC03	Trastuzumab
L01XC13	Pertuzumab

L01XC14	Trastuzumab-Emtansin
L01XE07	Lapatinib (metastatic disease)
L01EH02	Neratinib
**OPS breast surgery**	
5870, 5872, 5874, 5877, 5879	Breast surgery
54011%	Axillary sentinel node biopsy
54021%	Axillary lymphonodectomie
54040%, 54061%, 54070%	Axillary lymphonodectomie without surgery of the breast
**OPS biopsies and paracentesis**	
1844%	Diagnostic puncture of the pleural cavity
1845%	Diagnostic liver puncture
143%	Biopsy of the respiratory system
1481%	Biopsy of the bones
1441%	Needle-biopsy of the liver and the pancreas
8152%	Therapeutic puncture of thoracic organs
8153%	Paracentesis
8154%	Therapeutic puncture of abdominal organs

% indicates that each number between 0 – 9 could be selected

Supplementary Table S3: Match quality

Birth year difference	N
-2	1
-1	2
0	55714
1	17
3	1
5	1
7	2

Supplementary Table S4: **5- and 10-year overall survival rates stratified after disease stage**

	5 year survival			10 year survival			
Stage	N	Mean	SD	N	Mean	SD	p value
Control	55738	0.834	0.002	55738	0.677	0.004	reference
A early	18892	0.840	0.003	18892	0.663	0.007	n.s
B lymph nodes	4732	0.793	0.007	4732	0.566	0.015	$p < 0.001$
C metastases	4245	0.354	0.008	4245	0.209	0.010	$p < 0.001$

SD = Standard deviation

Supplementary Table S5: **5-year overall survival rates stratified after disease stage and estimated tumor subtype**

		Breast Cancer			Control			
Tumor biology	Stage	N	Mean survival	SD survival	N	Mean survival	SD survival	p value
HR+/HER2+	A early	1182	0.930	0.009	2364	0.948	0.005	$p < 0.01$
HR+/HER2-	A early	13542	0.867	0.003	27084	0.847	0.002	$p < 0.01$
HR-/HER2+	A early	472	0.896	0.016	944	0.931	0.009	n.s
HR-/HER2-	A early	3696	0.705	0.008	7392	0.785	0.005	$p < 0.001$
HR+/HER2+	B lymph nodes	439	0.888	0.017	878	0.931	0.010	$p < 0.001$
HR+/HER2-	B lymph nodes	3581	0.821	0.007	7162	0.870	0.004	$p < 0.001$
HR-/HER2+	B lymph nodes	158	0.792	0.035	316	0.921	0.017	$p < 0.001$
HR-/HER2-	B lymph nodes	554	0.541	0.023	1108	0.790	0.014	$p < 0.001$
HR+/HER2+	C metastases	309	0.532	0.032	618	0.881	0.014	$p < 0.001$
HR+/HER2-	C metastases	2644	0.418	0.011	5288	0.770	0.006	$p < 0.001$
HR-/HER2+	C metastases	187	0.494	0.039	374	0.869	0.019	$p < 0.001$
HR-/HER2-	C metastases	1105	0.127	0.011	2210	0.678	0.011	$p < 0.001$

Supplementary Table S6: 5-year distant recurrence-free survival rates stratified after disease stage and estimated tumor subtype

Stage	Tumor biology	N	Mean survival	SD survival	p value
A early	HR+/HER2+	1182	0.867	0.011	reference
A early	HR+/HER2-	13542	0.914	0.003	p < 0.001
A early	HR-/HER2+	472	0.845	0.019	n.s
A early	HR-/HER2-	3696	0.879	0.006	n.s
B lymph nodes	HR+/HER2+	439	0.809	0.021	reference
B lymph nodes	HR+/HER2-	3581	0.821	0.007	n.s
B lymph nodes	HR-/HER2+	158	0.699	0.039	p < 0.01
B lymph nodes	HR-/HER2-	554	0.659	0.023	p < 0.001
